# NTBI levels in C282Y homozygotes after therapeutic phlebotomy

**DOI:** 10.1002/jha2.507

**Published:** 2022-07-27

**Authors:** Eleanor Ryan, Keith Mulready, Erwin Wiegerinck, Jennifer Russell, Dorine W. Swinkels, Stephen Stewart

**Affiliations:** ^1^ Liver Centre Mater Misericordiae University Hospital Dublin Ireland; ^2^ Department of Biochemistry and Diagnostic Endocrinology Mater Misericordiae University Hospital Dublin Ireland; ^3^ Laboratory of Genetic, Endocrine and Metabolic Diseases, Department of Laboratory Medicine Radboud University Medical Centre Nijmegen The Netherlands

**Keywords:** ferritin, hereditary haemochromatosis, non‐transferrin bound iron, phlebotomy, transferrin saturation

## Abstract

C282Y homozygotes exposed to sustained elevated transferrin saturation (TS) may develop worsening clinical symptoms. This might be related to the appearance of non‐transferrin bound iron (NTBI) when TS≥50% and labile plasma iron (LPI) when TS levels reach 75–80%. In this study, NTBI levels were examined in 219 randomly selected untreated and treated C282Y homozygotes. Overall, 161 of 219 had TS ≥ 50%, 124 of whom had detectable NTBI (≥0.47 µM, 1.81 µM [0.92–2.46 µM]) with a median serum ferritin 320 µg/L (226–442 µg/L). Ninety of 219 homozygotes had TS ≥ 75%, and all had detectable NTBI (2.21 µM [1.53–2.59 µM] with a median ferritin 338 µg/L [230–447 µg/L]). Of 125 homozygotes who last had phlebotomy ≥12 months ago (42 months [25–74 months], 92 had TS levels ≥ 50%, and 70 of these had NTBI ≥ 0.47 µM (2.06 µM [1.23–2.61µM]). Twenty‐six of these 70 had a normal ferritin. Fifty‐five of 125 had TS ≥ 75%, and NTBI was detected in all of these (2.32 µM [1.57–2.77 µM]) with a median ferritin 344 µg/L (255–418 µg/L). Eighteen of these 55 had a normal ferritin. In summary, NTBI is frequently found in C282Y homozygotes with TS ≥ 50%. Furthermore, C282Y homozygotes in the maintenance phase often have TS ≥ 50% together with a normal ferritin. Therefore, monitoring the TS level during the maintenance phase is recommended as an accessible clinical marker of the presence of NTBI.

## 1 INTRODUCTION

In Ireland, 93% of individuals with hereditary haemochromatosis (HH) are homozygous for the C282Y mutation in the *HFE* gene [1, 2]. C282Y homozygotes are deficient in the liver‐derived hormone hepcidin, leading to increased intestinal iron absorption and systemic iron overload [3, 4, 5, 6].

Phlebotomy treatment lowers hepcidin by normalising serum ferritin levels, but phlebotomy can also increase iron absorption, further lowering hepcidin via erythropoiesis [4, 7, 8, 9]. However, normalising the ferritin level does not guarantee that transferrin saturation (TS) levels are under control as TS levels are frequently elevated in the maintenance phase despite a normal ferritin [10, 11]. Sustained exposure to TS ≥ 50% is associated with a worsening of arthritis in HH individuals on long‐term maintenance and may be related to increased levels of non‐transferrin bound iron (NTBI) [12, 13, 14, 15].

When TS levels exceed 75%–80%, the redox active component of NTBI, labile plasma iron (LPI) is found [16, 17]. LPI is highly reactive and damages organs through the formation of free radicals and lipid peroxidation [11, 15]. NTBI induced by sustained exposure to high TS levels may also predispose individuals to morbidities associated with aging such as stroke, diabetes and joint problems [18].

We have previously examined hepcidin and NTBI in a cohort comprising untreated non‐iron loaded and iron loaded C282Y homozygotes, compound heterozygotes (heterozygous for C282Y and H63D) and others with less at risk of iron overload genotypes. We found that C282Y homozygotes had significantly lower hepcidin levels and significantly higher NTBI levels compared to those with the ‘less at risk of iron overload’ genotypes [6]. We also reported a strong correlation of TS with NTBI [6].

While studies have shown that TS levels are often high in the maintenance phase even in the presence of a normal ferritin, there is a lack of information regarding NTBI levels following therapeutic phlebotomy. Consequently, the aim of this study was to examine NTBI in untreated and treated C282Y homozygotes and to look at its relationship with TS, hepcidin and other iron indices. We also sought to explore the relationship of NTBI with obesity, alcohol and joint pain.

## 2 MATERIALS AND METHODS

### 2.1 Participants

Fasting serum samples were randomly collected (8.30 am to 11 am) between February 2017 and November 2019, from 136 male and 83 female C282Y homozygous patients attending the Liver Centre and stored at −80°C. Patients were identified via family screening or presented with symptoms/raised iron indices and were diagnosed from 1986 to 2019. Phlebotomy treatment was initiated when the serum ferritin was raised (≥300 µg/l for males and ≥ 200µg for females) and entailed weekly venesection until the ferritin was in the normal range (<300 µg/l for males and <200 µg for females, [16]) with a target of ≤100 µg/L. Once depleted, ferritin levels are monitored to ensure they remain within the normal reference range before phlebotomy recommences (maintenance phase). The study received approval from the ethics committee of the Mater Misericordiae University Hospital, and informed consent was obtained from all participants.

Body mass index (BMI) was measured and participants completed a self‐administered questionnaire, from which information regarding alcohol consumption, joint pain and phlebotomy history were obtained. In Ireland, the low risk alcohol limit is <17 and <11 standard drinks per week for men and women, respectively (one standard drink contains 10 g pure alcohol). Additional phlebotomy information was obtained from the hospital record system.

### 2.2 Laboratory measurements

Serum iron, transferrin, glucose, C‐reactive protein (CRP), liver function tests, lipids, haemoglobin (Hgb) and serum ferritin were measured using routine methods. *HFE* genotyping was performed using LightCycler technology (Roche Diagnostics) with Genes‐4U toolsets (Ratiogen, AG).

Serum hepcidin was measured using the high sensitivity enzyme immunoassay ELISA kit (Hepcidin 25 (bioactive), EIA‐5782, DRG Diagnostics, Marburg, Germany). Thawed samples (one thaw only) were analysed for hepcidin between April and September 2019. Low and high controls provided in the kit were run with each calibration curve, and results were considered invalid if the assay did not fit the acceptable ranges of the low and high controls. The assay range is between 0.153 ng/ml and 81 ng/ml. The limit of detection is 0.304 ng/ml. NTBI was measured using a nitrilotriacetic (NTA) assay [19]. The lower limit of detection was <0.47 µM. For those samples that received a <0.47 µM value, a value of 0.46 was entered onto the SPSS database. NTBI was measured from October to December 2020.

### 2.3 Statistical analyses

All data were tested for normality using the Kolmogorov‐Smimov test. Non‐normal data are presented as the median (interquartile range [25th–75th percentile]), while normal data are presented as means ± standard deviation. Differences between groups were calculated using the non‐parametric Mann‐Whitney *U* test. Relationships between variables were assessed using Spearman r. Statistical evaluation was carried out using IBM SPSS for Windows, version 25 (IBM Corp., Armonk, NY, USA).

## 3 RESULTS

### 3.1 Baseline characteristics

The main characteristics of the study cohort are presented in Table 1. The 219 C282Y homozygotes were diagnosed between 1986 and 2019. For those who underwent phlebotomy treatment, the median end of treatment ferritin was 131 µg/L (96—168 µg/L) and 165 µg/L (130–200 µg/L) for males and females, respectively. Twenty‐seven males and 34 females never had phlebotomy treatment, while 21 males and 12 females had phlebotomy within the 12 months prior to the study, while 88 males and 37 females last had phlebotomy ≥12 months prior to the study. The 12‐month cut off point was chosen because iron absorption has been shown to be increased in the 12 months following phlebotomy treatment, and phlebotomy can further lower hepcidin via erythropoiesis [4, 7–9, 20].

**TABLE 1:** Characteristics of male and female C282Y homozygotes

Variables*	**Male**	**Female**	**Mann‐Whitney *U* test *p*‐value**
Age (years)	53.8 ± 14.1	52.7 ± 14.0	0.742
Serum iron (µmol/l)	33.9 ± 8.9	29.0 ± 8.4	<0.001
Transferrin (g/l)	1.92 ± 0.27	1.91 ± 0.37	0.658
Transferrin saturation (%)	75.4 (53.2–85.9)	59.6 (42.6–75.2)	<0.001
Hepcidin (ng/ml)	10.2 (7.1–14.9)	12.0 (6.3–14.9)	0.422
Serum ferritin (µg/l)	271 (160–397)	230 (73–333)	0.024
Hepcidin:ferritin ratio	0.042 (0.027–0.063)	0.059 (0.039–0.103)	<0.001
NTBI (µM)	0.95 (0.46–2.25)	0.53 (0.46–1.32)	0.009
Hgb (g/dl)	15.6 (14.6–16.2)	14.3 (13.7–14.9)	<0.001
Bilirubin (µmol/L)	13 (10–17)	10 (8–13)	<0.001
ALT (I.U./L)	28.0 (21–39.3)	20 (16–30)	<0.001
γGT (I.U./L)	30 (22–44.0)	20 (15–27)	<0.001
AST (I.U./L)	29 (25–36)	26 (22–31)	0.001
Alkp (I.U./L)	74 (64–86)	70 (59‐90)	0.326
Glucose (mmol/L)	4.9 (4.6–5.3)	4.8 (4.4–5.2)	0.148
C‐reactive protein (CRP) (mg/L)	2.0 (1.0–3.0)	2.0 (1.0–4.0)	0.344
Cholesterol (mmol/L)	4.8 ± 0.9	5.0 ± 0.8	0.132
Triglycerides (mmol/L)	1.31 (0.91–1.77)	1.18 (0.84–1.57)	0.059
HDL (mmol/L)	1.21 (1.06–1.44)	1.50 (1.31–1.81)	<0.001
LDL (mmol/L)	2.90 (2.20–3.40)	2.85 (2.20–3.50)	0.892
Joint pain (no/yes)	31/41	19/20	
Body mass index (BMI)	27.0 (24.5–29.6) (*n* = 84)	26.3 (23.5–29.3) (*n* = 52)	0.306
Alcohol (std drinks/week)	7.5 (2–15) (*n* = 110)	3.5 (0.75–7.3) (*n* = 66)	0.001
Phlebotomy	(*n*)	(*n*)	
Never had phlebotomy	27	34	
<12 months since last phlebotomy	21	12	
≥12 months ago since last phlebotomy	88	37	

* All values are those determined at the time the sample was taken and are not diagnostic values. Data were tested for normality using the Kolmogorov‐Smimov test. Non‐normal data are presented as the median (25th–75th percentile), while normal data are presented as means ± standard deviation. Differences between groups were calculated using the non‐parametric Mann‐Whitney *U* test. Data refer to the date on which the blood sample was taken. Note: 49 males and 40 females had NTBI <0.47 µM (not detected). A value of 0.46 µM represents undetected NTBI.

### 3.2 Hepcidin levels in non‐iron loaded and iron loaded male and female C282Y homozygotes

**FIGURE 1:**
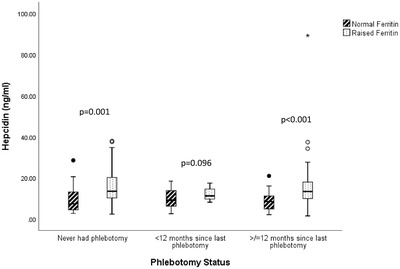
Boxplots of hepcidin levels in C282Y homozygotes according to phlebotomy status. A normal serum ferritin was defined as a serum ferritin <300 µg/l for males and <200 µg for females. The line in the middle of the boxplot is the median. The top and bottom box lines show the 75th percentile and 25th percentile. The whiskers show the maximum and minimum values. Outliers (circles) are at least 1.5 box lengths from the median, while extremes (asterisks) are at least 3 box lengths from the median

**FIGURE 2:**
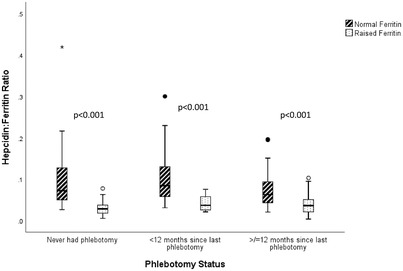
Boxplots of hepcidin:ferritin ratios in C282Y homozygotes according to phlebotomy status. A normal serum ferritin was defined as a serum ferritin <300 µg/l for males and <200 µg for females. The line in the middle of the boxplot is the median. The top and bottom box lines show the 75th percentile and 25th percentile. The whiskers show the maximum and minimum values. Outliers (circles) are at least 1.5 box lengths from the median, while extremes (asterisks) are at least 3 box lengths from the median

C282Y homozygotes with a raised ferritin regardless of phlebotomy status had higher hepcidin levels and lower hepcidin : ferritin ratios compared to those with a normal ferritin (Figures 1 and 2).

Overall, hepcidin was significantly higher in C282Y homozygotes with a raised ferritin (for their gender) (*n* = 109), 13.10 ng/ml (10.00–18.22 ng/ml) compared to those with a normal ferritin (*n* = 110) 8.30 ng/ml (4.82–11.86 ng/ml), *p* < 0.001. The hepcidin : ferritin ratio was significantly lower in C282Y homozygotes with a raised ferritin, 0.032 (0.021–0.048) compared to those with a normal ferritin (0.068 [0.047–0.109]), *p* < 0.001.

### 3.3 TS and NTBI levels in male and female C282Y homozygotes

**FIGURE 3:**
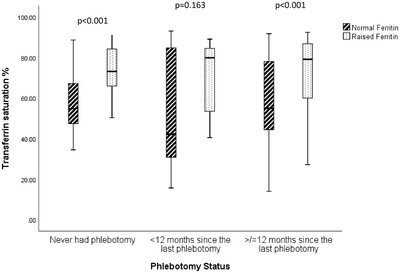
Boxplots of transferrin saturation levels in C282Y homozygotes according to phlebotomy status. A normal serum ferritin was defined as a serum ferritin <300 µg/l for males and <200 µg for females. The line in the middle of the boxplot is the median. The top and bottom box lines show the 75th percentile and 25th percentile. The whiskers show the maximum and minimum values

C282Y homozygotes with a raised ferritin regardless of phlebotomy status had higher TS levels compared to those with a normal ferritin (Figure 3).

Male and female C282Y homozygotes with NTBI ≥ 0.47 µM (the level at which NTBI becomes detectable), regardless of phlebotomy status, or whether the ferritin was normal or raised, had higher TS levels (Tables 2 and 3).

**TABLE 2:** Characteristics and serum iron indices according to a normal or elevated ferritin, phlebotomy status and NTBI level in male C282Y homozygotes

		Never had phlebotomy			<12 months since last phlebotomy			≥12 months since last phlebotomy		
		Overall	Normal Ferritin	Raised Ferritin	Overall	Normal Ferritin	Raised Ferritin	Overall	Normal Ferritin	Raised Ferritin
		*n = 7*	*n = 4*	*n = 3* *	*n = 9*	*n = 9*	*n = 0*	*n = 33*	*n = 26*	*n = 7*
NTBI < 0.47 (μM) (n = 49)	Age (years)	56.1 ± 15.9	55.8 ± 15.1	56.7 ± 20.3	53.9 ± 15.4	53.9 ± 15.4		58.0 ± 12.7	55.4 ± 12.4	66.6 ± 10.8
	Serum iron (μmol/l)	30.1 ± 5.5	27.7 ± 5.2	33.3 ± 4.8	19.0 ± 6.2	19.0 ± 6.2		26.7 ± 6.6	25.8 ± 6.6	30.1 ± 6.31
	Trans Sat (%)	61.3 (54.1–68.6)	54.5 (41.6–65.3)	66.7 (61.3–	38.0 (23.0–44.0)	38.0 (23.0–44.0)		48.3 (43.5–56.7)	47.1 (41.7–54.0)	57.1 (53.1–68.9)
	Transferrin (g/l)	1.98 ± 0.28	2.01 ± 0.35	1.94 ± 0.20	2.15 ± 0.25	2.15 ± 0.25		2.07 ± 0.26	2.10 ± 0.26	1.94 ± 0.21
	Hepcidin (ng/ml)	13.4 (4.9–20.6)	9.2 (3.3–18.8)	19.1 (8.5–	9.2 (4.1–13.5)	9.2 (4.1–13.5)		9.2 (4.6–11.8)	7.1 (4.5–10.2)	16.8 (11.4–18.2)
	Ferritin (μg/l)	273 (74–922)	139 (61–256)	922 (418–	158 (38–218)	158 (38–218)		147 (63–258)	95 (55–186)	398 (313–536)
	Hepcidin:Ferritin Ratio	0.05 (0.02–0.07)	0.06 (0.05–0.09)	0.02 (0.02–	0.07 (0.06–0.12)	0.07 (0.06–0.12)		0.06 (0.04–0.10)	0.07 (0.04–0.11)	0.04 (0.03–0.04)
		*n = 20*	*n = 4*	*n = 16*	*n = 12*	*n = 7*	*n = 5*	*n = 55*	*n = 26*	*n = 29*
NTBI ≥ 0.47 (μM) (n = 87)	Age (years)	44.1 ± 14.0	39.5 ± 21.5	45.3 ± 12.2	53.4 ± 16.1	48.1 ± 14.6	60.8 ± 16.5	54.6 ± 13.0	55.3 ± 11.4	54.1 ± 11.4
	Serum iron (μmol/l)	38.8 ± 6.8	39.9 ± 6.4	38.5 ± 7.1	39.6 ± 5.9	40.3 ± 7.2	38.5 ± 3.8	38.0 ± 5.8	36.1 ± 5.7	39.8 ± 5.4
	Trans Sat (%)	82.9 (73.5–88.5)	83.7 (76.7–87.5)	82.7 (72.5–89.1)	85.5 (82.6–88.2)	85.9 (83.6–88.3)	85.0 (70.9–87.5)	82.2 (77.3–88.2)	78.7 (64.6–86.1)	85.8 (79.8–88.9)
	Transferrin (g/l)	1.84 ± 0.19	1.84 ± 0.21	1.85 ± 0.19	1.80 ± 0.22	1.78 ± 0.28	1.84 ± 0.12	1.83 ± 0.26	1.84 ± 0.30	1.83 ± 0.23
	Hepcidin (ng/ml)	13.2 (9.8–22.2)	7.6 (6.0–11.0)	16.2 (10.6–29.3)	9.9 (8.2–13.2)	9.9 (7.2–13.9)	10.0 (9.4–14.5)	10.2 (7.5–14.8)	9.6 (7.1–11.8)	11.8 (8.2–18.0)
	Ferritin (μg/l)	533 (313–976)	225 (206–275)	676 (382–1110)	263 (143–416)	207 (76–244)	439 (335–590)	310 (228–389)	224 (161–257)	388 (349–554)
	Hepcidin:Ferritin Ratio	0.03 (0.01–0.04)	0.03 (0.03–0.04)	0.03 (0.01–0.04)	0.04 (0.03–0.06)	0.06 (0.05–0.10)	0.03 (0.02–0.03)	0.04 (0.02–0.05)	0.04 (0.03–0.06)	0.03 (0.02–0.05)
	NTBI (μM)	1.38 (0.77–2.05)	1.25 (0.65–1.89)	1.44 (0.77–2.19)	2.31 (1.70–2.85)	2.37 (1.63–2.74)	2.26 (1.47–2.89)	2.06 (1.26–2.60)	1.62 (0.61–2.40)	2.44 (1.59–2.83)

Non normal data are presented as the median (interquartile range (IQR) (25^th^‐75^th^ percentile)) while normal data are presented as means ± standard deviation. Differences between groups were calculated using the non‐parametric Mann‐Whitney U test. * SPSS does not generate 75^th^ percentile for cohort of 3 individuals.

**TABLE 3:** Characteristics and serum iron indices according to a normal or elevated ferritin, phlebotomy status and NTBI level in female C282Y homozygotes

		**Never had phlebotomy**			**<12 months since last phlebotomy**			**≥12 months since last phlebotomy**		
		Overall	Normal ferritin	Raised ferritin	Overall	Normal ferritin	Raised ferritin	Overall	Normal ferritin	Raised ferritin
		*n = 16*	*n = 12*	*n = 4*	*n = 6*	n = 3*	*n = 3*	*n = 18*	*n = 7*	*n = 11*
NTBI < 0.47 (μM) (n = 40)	Age (years)	46.7 ± 12.7	46.5 ± 14.6	47.3 ± 5.0	55.3 ± 11.8	45.3 ± 8.3	61.3 ± 9.4	59.9 ± 10.1	60.1 ± 7.8	59.7 ± 11.6
	Serum iron (μmol/l)	27.6 ± 6.2	26.5 ± 7.0	27.4 ± 3.4	20.3 ± 3.6	19.2 ± 4.6	21.4 ± 2.9	23.5 ± 6.3	21.7 ± 6.6	24.8 ± 6.1
	Trans Sat (%)	51.5 (42.9–59.6)	48.5 (39.4–56.1)	64.2 (53.3–66.8)	40.4 (28.9–41.4)	30.8 (23.1–)	41.0 (40.5–)	46.8 (38.4–54.7)	45.0 (27.8–52.7)	50.4 (38.6–60.9)
	Transferrin (g/l)	2.02 ± 0.43	2.12 ± 0.45	1.73 ± 0.21	2.18 ± 0.36	2.38 ± 0.38	1.98 ± 0.21	1.95 ± 0.24	1.96 ± 0.28	1.95 ± 0.23
	Hepcidin (ng/ml)	11.1 (5.3–15.9)	9.6 (4.5–15.9)	12.3 (10.5–17.9)	13.7 (6.3–17.3)	6.3 (6.2–)	14.9 (12.5–)	13.1 (8.6–17.1)	7.9 (4.7–11.3)	14.6 (13.0–27.6)
	Serum iron (μmol/l)	27.6 ± 6.2	26.5 ± 7.0	27.4 ± 3.4	20.3 ± 3.6	19.2 ± 4.6	21.4 ± 2.9	23.5 ± 6.3	21.7 ± 6.6	24.8 ± 6.1
	Ferritin (μg/l)	155 (64–235)	132 (42–163)	336 (262–534)	157 (35–290)	40 (21–)	289 (49–)	256 (103–321)	68 (65–121)	291 (260–379)
	Hepcidin:Ferritin Ratio	0.09 (0.05–0.13)	0.11 (0.07–0.14)	0.04 (0.03–0.05)	0.11 (0.06–0.19)	0.16 (0.15–)	0.06 (0.05–)	0.07 (0.05–0.11)	0.09 (0.07–0.12)	0.05 (0.04–0.10)
		*n = 18*	*n = 7*	*n = 11*	*n = 6*	n = 2*	*n = 4*	*n = 19*	*n = 3*	*n = 16*
NTBI ≥ 0.47 (μM) (n = 43)	Age (years)	44.4 ± 14.8	36.1 ± 13.4	49.6 ± 13.6	57.0 ± 8.2	58.0 ± 1.4	56.5 ± 10.5	57.3 ± 14.8	66.3 ± 11.6	55.6 ± 15.0
	Serum iron (μmol/l)	30.8 ± 4.6	30.3 ± 6.0	31.1 ± 3.8	33.6 ± 9.2	29.7 ± 16.9	35.6 ± 5.6	35.5 ± 9.5	33.7 ± 16.0	35.8 ± 8.5
	Trans Sat (%)	66.2 (62.2–84.7)	64.3 (50.7–86.2)	73.1 (62.4–84.2)	81.3 (67.2–85.1)	59.5 (30.4–)	81.3(79.6–83.5)	79.7 (64.3–86.6)	70.4 (28.2–)	80.0 (65.1–87.6)
	Transferrin (g/l)	1.73 ± 0.33	1.84 ± 0.40	1.67 ± 0.28	1.78 ± 0.30	2.01 ± 0.30	1.67 ± 0.25	1.92 ± 0.41	2.22 ± 0.49	1.86 ± 0.39
	Hepcidin (ng/ml)	10.5 (4.5–13.7)	4.7 (3.6–10.1)	12.8 (8.1–14.9)	10.5 (6.8–14.8)	9.4 (2.6–)	10.5 (8.6–13.5)	12.6 (9.1–16.9)	5.8 (3.9–)	13.0 (10.2–17.0)
	Ferritin (μg/l)	242 (66–371)	62 (24–71)	335 (253–443)	208 (60–250)	49 (26–)	218 (205–305)	295 (248–424)	68 (20–)	333 (273–441)
	Hepcidin:Ferritin Ratio	0.05 (0.03–0.08)	0.09 (0.05–0.22)	0.03 (0.02–0.05)	0.06 (0.04–0.13)	0.17 (0.10–)	0.05 (0.03–0.06)	0.04 (0.02–0.08)	0.20 (0.09–)	0.04 (0.02–0.06)
	NTBI (μM)	0.96 (0.80–1.37)	0.83 (0.61–1.17)	1.00 (0.83–1.81)	2.21 (1.73–2.54)	1.86 (0.66–)	2.21 (2.11–2.34)	1.34 (0.69–2.60)	0.69 (0.47–)	1.87 (0.76–2.75)

Non normal data are presented as the median (interquartile range (IQR) (25^th^‐75^th^ percentile)) while normal data are presented as means ± standard deviation. Differences between groups were calculated using the non‐parametric Mann‐Whitney U test. * SPSS does not generate 75^th^ percentile for cohorts of 2 or 3 individuals.

Overall, TS levels were significantly higher in those with NTBI ≥ 0.47 µM (*n* = 130), 82.3% (72.9%–87.0%) compared to those with NTBI < 0.47 µM (*n* = 89), 47.6% (40.4%–56.4%), *p* < 0.001.

In the cohort overall (*n* = 219), 161/219 (74%, 105 males and 56 females) had TS ≥ 50%, 124 of whom (77%) had NTBI ≥ 0.47 µM (1.81 µM [0.92–2.46µM]) with a median serum ferritin 320 µg/L (226–442 µg/L). Ninety of 219 (41%, 68 males and 22 females) had TS ≥ 75% with a median serum ferritin 338 µg/L (230–447 µg/L), and all had NTBI ≥ 0.47 µM (2.21 µM [1.53–2.59 µM]). Ten per cent of those with a TS < 50% (6 of 58) and 31% of those with a TS < 75% (40 of 129) had NTBI ≥ 0.47 µM.

### 3.4 TS levels of ≥50% and ≥75% and the presence of NTBI in those who last had phlebotomy ≥12 months ago

In the cohort of 125 male and female C282Y homozygotes who last had phlebotomy ≥12 months ago (42.0 months [25–74 months]), 92 (74%, 67 males and 25 females) had TS levels ≥50% and 70 of these (76%) had NTBI ≥ 0.47 µM (2.06 µM [1.23–2.61 µM]). Thirty‐seven per cent of these (26/70, 24 males and two females) had a normal ferritin. Forty‐four per cent (55/125, 44 males and 11 females) had TS ≥ 75%, and all had NTBI ≥ 0.47 µM (2.32µM [1.57–2.77 µM]). Thirty‐three per cent (18/55) of these (17 males and one female) had a normal ferritin.

### 3.5 Iron parameters, obesity, alcohol and joint pain

BMI was available for 84 males and 52 females. Seventeen of 84 males (20%) and 10 of 52 females (19%) were obese (BMI ≥ 30). No significant differences in TS, hepcidin, ferritin or hepcidin : ferritin ratio were noted between those who were obese and those who were not obese nor when analysed according to phlebotomy status. But, TS levels tended to be lower in obese homozygotes (males, *p* = 0.070; females, *p* = 0.057). Forty‐seven per cent (8/17) obese males had NTBI ≥ 0.47 µM compared to 67.2% (45/67) of the non‐obese cohort, while 25% (4/10) obese females had NTBI ≥ 0.47 µM compared to 60% (25/42) of the non‐obese cohort.

110 males and 66 females responded to the questionnaire regarding alcohol intake. Twenty‐one of 110 males (19%) and eight of 66 females (12%) had an alcohol intake above that recommended for their gender. Serum iron levels were significantly higher in males who drank ≥17 units alcohol per week (37.6 µmol/l ± 7.6µmol/l) compared to those who drank <17 units per week (33.0 µmol/l ± 8.6 µmol/l), *p* = 0.009. No other significant differences were noted. But, TS levels tended to be higher in those who drank alcohol at levels beyond that recommended (males, *p* = 0.065; females, *p* = 0.377). Eighty‐one per cent (17/21) of males who drank ≥17 units per week had NTBI ≥ 0.47 µM compared to 61% (54/89) of those who drank <17 units per week. Fifty per cent (4/8) of females who drank ≥11 units per week had NTBI ≥ 0.47 µM compared to 52% (30/58) of females who drank <11 alcohol units per week.

One hundred and eleven individuals responded to the questionnaire regarding joint pain, 41 of 72 (57%) of males and 20 of 39 (51%) of females responded yes to the query about whether they had joint pain (Table 1). Those who reported joint pain were older (55.5 ± 11.8 years vs. 50.0 ± 14.9 years, *p* = 0.059 for males; 60.7 ± 10.3 years vs. 46.0 ± 16.2 years, *p* = 0.004 for females). No other statistical differences were noted for serum iron, TS, ferritin or hepcidin levels in those males/females who reported joint pain compared to those who did not. Twenty‐six of 41 (63%) males and 10 of 20 (50%) females who reported having joint pain had NTBI ≥ 0.47µM.

### 3.6 Correlations

**FIGURE 4:**
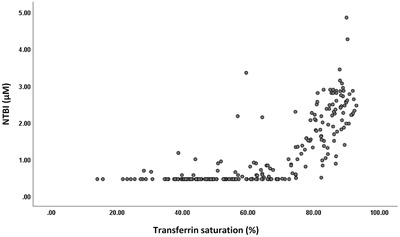
Scatterplot of NTBI versus transferrin saturation for the entire cohort. Spearman correlation *r* = 0.839, *p* < 0.001, *n* = 219

In the cohort overall, NTBI correlated strongly with TS (Spearman *r* = 0.839, *p* < 0.001; Figure 4) and serum iron (Spearman *r* = 0.747, *p* < 0.001) and less strongly with ferritin (Spearman *r* = 0.429, *p* < 0.001). Ferritin also correlated with hepcidin (Spearman *r* = 0.541, *p* < 0.001), TS (Spearman *r* = 0.512, *p* < 0.001) and serum iron (Spearman *r* = 0.426, *p* < 0.001).

## 4 DISCUSSION

In this study of C282Y homozygotes, NTBI was present in 77% of patients who had TS ≥ 50% and in 100% of patients with TS ≥ 75% irrespective of treatment status. Previous studies have suggested that it can be taken for granted that the potentially harmful component of NTBI, LPI is present at TS levels of 70%–90%, so LPI is also most likely present in the sera of our C282Y homozygotes [15, 16, 17, 21].

Thirty‐one per cent of those with a TS level < 75% and 10% of those with a TS level < 50% had NTBI ≥ 0.47 µM demonstrating that NTBI can appear in the absence of fully saturated transferrin, which is consistent with other studies [22, 23, 24].

In agreement with other reports, TS was found to be frequently elevated in the maintenance phase [17, 18]. In the cohort who last had phlebotomy at least a year prior to the study, 74% had TS ≥ 50%, and 76% of these had detectable NTBI, while 44% had TS ≥ 75%, and all had detectable NTBI. This represents a significant proportion of C282Y homozygotes who may have been exposed to sustained elevated TS and NTBI, and at levels of TS ≥ 75%, prolonged exposure to LPI. Sustained exposure to TS ≥ 50% for more than 6 years and to TS ≥ 75% for more than 8 months has been associated with a worsening of metacarpophalangeal and proximal interphalangeal joint symptoms, which are specifically associated with HH [12]. The current study did not look at sustained exposure to high TS levels but it is hoped that further studies will address this.

Thirty‐seven per cent and 33% of those with TS ≥ 50% and TS ≥ 75%, respectively, who last had phlebotomy at least a year prior to the study had a normal ferritin for their gender demonstrating that a normal ferritin is no guarantee that the TS level is under control, which is in agreement with other studies that have found elevated TS levels in the maintenance phase despite normal body iron stores [10, 11].

Our data might suggest that because of the risk of a sustained exposure to high TS levels and thus the probability of the LPI being present, that phlebotomy treatment should ensure that TS is maintained < 50%. However, this must be tempered with the fact that excessive phlebotomy treatment may result in iron deficiency [18, 25]. An alternative approach may involve the use of therapies that correct hepcidin deficiency, which would be beneficial in the maintenance phase to control TS and thus NTBI and consequently ameliorate the long‐term complications in HH patients [11, 18, 26].

Our data confirm that ferritin levels play a significant role in determining hepcidin levels regardless of phlebotomy status demonstrating that C282Y homozygotes retain some ability to up‐regulate hepcidin in response to iron overload [4, 9]. However, the significantly lower hepcidin : ferritin ratios in the iron loaded cohorts show that this response is entirely inadequate. Clearly, while phlebotomy results in the removal of excess iron, this does not restore the inadequate physiological response of hepcidin to iron overload in C282Y homozygotes [11].

Obese individuals had higher hepcidin and lower TS levels (although not significantly), which may be consistent with low grade inflammation that is associated with a high BMI [27, 28, 29, 30]. We have previously found significantly higher hepcidin levels in overweight male C282Y homozygotes indicating perhaps that hepcidin is up‐regulated in response to low grade inflammation, which may partly protect against the inadequate production of hepcidin (arising from the mutation in HFE) and thereby modulating phenotypic expression of HH [6]. The observation that a lower percentage of obese homozygotes had detectable NTBI is also in keeping with the lower TS noted in this cohort and a previously described decreased iron burden in overweight female C282Y homozygotes [28, 29].

Males whose alcohol intake was ≥ 17 standard units per week had higher TS and NTBI levels (although again not significantly) which have previously been reported in individuals who drink alcohol in excess and are associated with a greater incidence of liver cirrhosis [23, 31]. Ferritin levels also tended to be higher in those who drank alcohol to excess in agreement with previous reports [32]. In view of the double assault of C282Y homozygosity and alcohol on the liver, further investigations to clarify the pathogenic role of NTBI are warranted in this cohort.

Those who reported joint pain were significantly older, but our study of joint pain in HH is limited because of our reliance on self‐administered questionnaires and our lack of information on the type of joint pain as has been previously highlighted [12, 33]. In addition, we have only a one point measurement of NTBI, and the development of joint pain may arise from long‐term exposure to high iron levels.

While our data demonstrate that NTBI is always present at TS levels ≥ 75%, the clinical relevance of this is largely unknown. A single measurement of NTBI only represents the previous 24–48 h so repeated measurements are required to ascertain toxicity potential and indeed the exposure time to NTBI [17, 34]. Furthermore, the application of clinical assays for NTBI is limited because of the lack of standardisation between the methods used. However, high TS levels can be used as a surrogate marker for the presence of NTBI at TS 70%–90% [16, 17].

Despite the fact that high TS levels are frequently found in the maintenance phase, international guidelines do not currently recommend its measurement in the follow‐up maintenance phase [35, 36]. However, in light of the data presented in this study demonstrating that high TS is associated with the appearance of NTBI, together with data that have shown that sustained exposure to high TS levels is associated with increased reports of fatigue and joint symptoms, it may be advisable to include the measurement of TS during the maintenance phase as has recently been recommended [37].

In conclusion, we have found raised NTBI levels in HH patients with raised TS and normal ferritins, the impact of which is unclear. Further studies are required to delineate the clinical significance of NTBI (in particular the clinical significance of sustained exposure to LPI) as the presence of NTBI has been associated with cardiovascular disease, diabetes, arthritis and an increased risk of cirrhosis, which are all elements of the HH clinical picture.

## CONFLICT OF INTEREST

The authors declare no competing financial interest. DWS is an employee of Radboudumc that offers analysis of iron biomarkers and reference material for a fee for service via its hepcidinanalysis.com initiative.

## ETHICS STATEMENT

The study received approval from the ethics committee of the Mater Misericordiae University Hospital, and informed written consent was obtained from all participants.

## AUTHOR CONTRIBUTIONS

E R designed the study, performed the hepcidin assay, analysed the data and wrote the paper. K M performed the hepcidin assay. E W performed NTBI measurements. J R performed HFE testing. S S and D W S reviewed the data and critically revised the paper.

## Data Availability

The data that support the findings of this study are available from the corresponding author upon reasonable request.
